# Scraping nasal cytology in the diagnostics of rhinitis and the comorbidities

**DOI:** 10.1038/s41598-022-18734-3

**Published:** 2022-08-25

**Authors:** Dorota Myszkowska, Monika Bazgier, Sara Brońska, Karol Nowak, Joanna Ożga, Aleksandra Woźniak, Andrzej Stanisz, Joanna Szaleniec

**Affiliations:** 1 Department of Clinical and Environmental Allergology, Jagiellonian University Medical College, Botaniczna 3, 31-503 Kraków, Poland; 2 Department of Otolaryngology, Jagiellonian University Medical College, Jakubowskiego 2, 30-688 Kraków, Poland; 3 Department of Bioinformatic and Telemedicine, Jagiellonian University Medical College, Medyczna 7, 30-688 Kraków, Poland

**Keywords:** Cell biology, Medical research

## Abstract

Nasal scraping cytology is a non-invasive tool used in the diagnostics of allergic and non-allergic rhinitis. The study aimed to analyze to what extent the cytological picture of the nasal mucosa coincides with the diagnosis of a given disease, taking into account the content of eosinophils. Retrospective analysis of the cytograms performed in 842 patients was carried out in relation to the disease entities and the content of eosinophils. Significant relationship between the Epith:Infl ratio and the four groups of diseases (Chi^2^ = 9.6488; p = .014) was confirmed. The more intensive inflammation was found, the higher percentage of patients had manifested the increased level of eosinophils (> 1% in the inflammatory cells). The value of 20% of eosinophils in all counted cells corresponds to around 45% of eosinophils in the inflammatory cells in patients with the evident inflammatory picture. Allergic rhinitis presents a different cytological picture regarding the eosinophilic reaction against the background of the inflammation process: the higher degree of inflammation observed, the lower amount of eosinophils detected, with the exception of allergic rhinitis provoked by pollen allergens.

## Introduction

Nasal cytology is a non-invasive tool that belongs to the cytological tests used in the diagnosis of respiratory tract diseases^1–3^, especially in the diagnostics and treatment of allergic and non-allergic rhinitis^4^. The cellular material collection includes brushing, scraping, nasal lavage, nasal blown secretion, mainly used in small children. Compared to the other methods, exfoliative (scraping) cytology shows the information about the live epithelial cells, being an advantage over nasal lavage, although the sampled area is relatively smaller^1,5,6^. In a normal cytogram, the cells of respiratory epithelium predominate, including cylindrical cells, mainly ciliated, and secretory (goblet) cells. Moreover, squamous epithelium can be observed in a very low quantity, while basal cells should not be present in cytological smears, apart from the intensive epithelium renewal^6,7^. In the intercellular spaces, the presence of neutrophils and few lymphocytes is possible, while in the physiological state eosinophils and metachromatic cells (mast cells/basophils) should not appear^1,5,6^.

Exfoliative cytology is an important diagnostic test used mainly in otolaryngology, especially in rhinology and in allergology, to evaluate the type of inflammatory cells in the nasal mucosa. The absence of bacterial or fungal cells in the cytological material indicates a non-infectious state^4^, while in infectious bacterial rhinitis and sinusitis, an increased content of neutrophils is usually observed, with intra- and extracellular bacteria^1^. The advantage of cytological examination is the possibility of assessing morphological changes in epithelial cells caused by a viral infection, which is confirmed by the cytopathic effect of viruses on the nasal mucosa^8^ presents even in the case of SARS-CoV-2 infection^9^.

Many authors emphasize the importance of cytology as one of the methods of indirect inference about tissue eosinophilia, important from the diagnostician point of view^1,4,10^. In case of uncertainty of allergological diagnosis, when the history is not consistent with the results of skin prick tests (SPT) or the concentration of specific IgE, the increased percentage of eosinophils in the cytological smear suggests the need for a nasal provocation test, which is a definitive test indicating entopy, i.e. local, allergic inflammation limited to the nasal mucosa, also known as "local atopy"^11–13^. An increase in the share of eosinophils also occurs in untreated allergic rhinitis (AR), non-allergic eosinophilic rhinitis (NARES), which may coincide with local allergic rhinitis (LAR) and in eosinophilic polyps^14,15^. It was reported that an increased content of eosinophils is observed in both intermittent and persistent AR, diagnosed according to the ARIA 2001 guidelines^16,17^.

On the other hand, the presence of an increased influx of neutrophils, according to some authors, is not of a great diagnostic value, as their presence can be observed, for example, in even 79% of asymptomatic school-age children^18^. However, the correct interpretation of the participation of all inflammatory cells in the cytogram allows for accurate phenotyping of non-allergic rhinitis, especially chronic rhinitis referred to in the literature as "cellular"^10,16^. Non-allergic rhinitis with neutrophils (NARNE) is characterized by a massive infiltration of neutrophils constituting more than 50% of total nasal cells, without bacteria occurrence^1,5^. Two types of non-allergic rhinitis of joined different cells infiltration are also distinguished, as non‐allergic rhinitis eosinophilic mast cell syndrome (NARESMA), in addition to the important eosinophilic infiltrate, in which a mast cell component (> 10% of total nasal cells) is detectable and non‐allergic rhinitis with mast cells (NARMA) characterized by the isolated mast cell infiltration (> 10% of total nasal cells). It is stressed that there is a need for further studies on the above-mentioned syndromes to determine the appropriate procedure to interpret the cytological tests in relation to the clinical picture of the patients and to introduce an effective treatment to avoid any complications^1,19–21^.

Despite some limitations indicated in the literature, such as a need to standardize the criteria of results interpretation, cytology is undoubtedly a diagnostic tool worth considering in many clinical aspects. Based on the authors’ experience in the analysis of a great pool of cytological smears in patients diagnosed with different diseases of upper respiratory tract, we aimed our study at checking to what extent the cytological picture of the nasal mucosa coincides with the diagnosis of a given disease, taking into account the intensity of inflammation and the content of eosinophils.

## Results

### Diagnosis versus the Epith:Inf ratio

**Figure 1:**
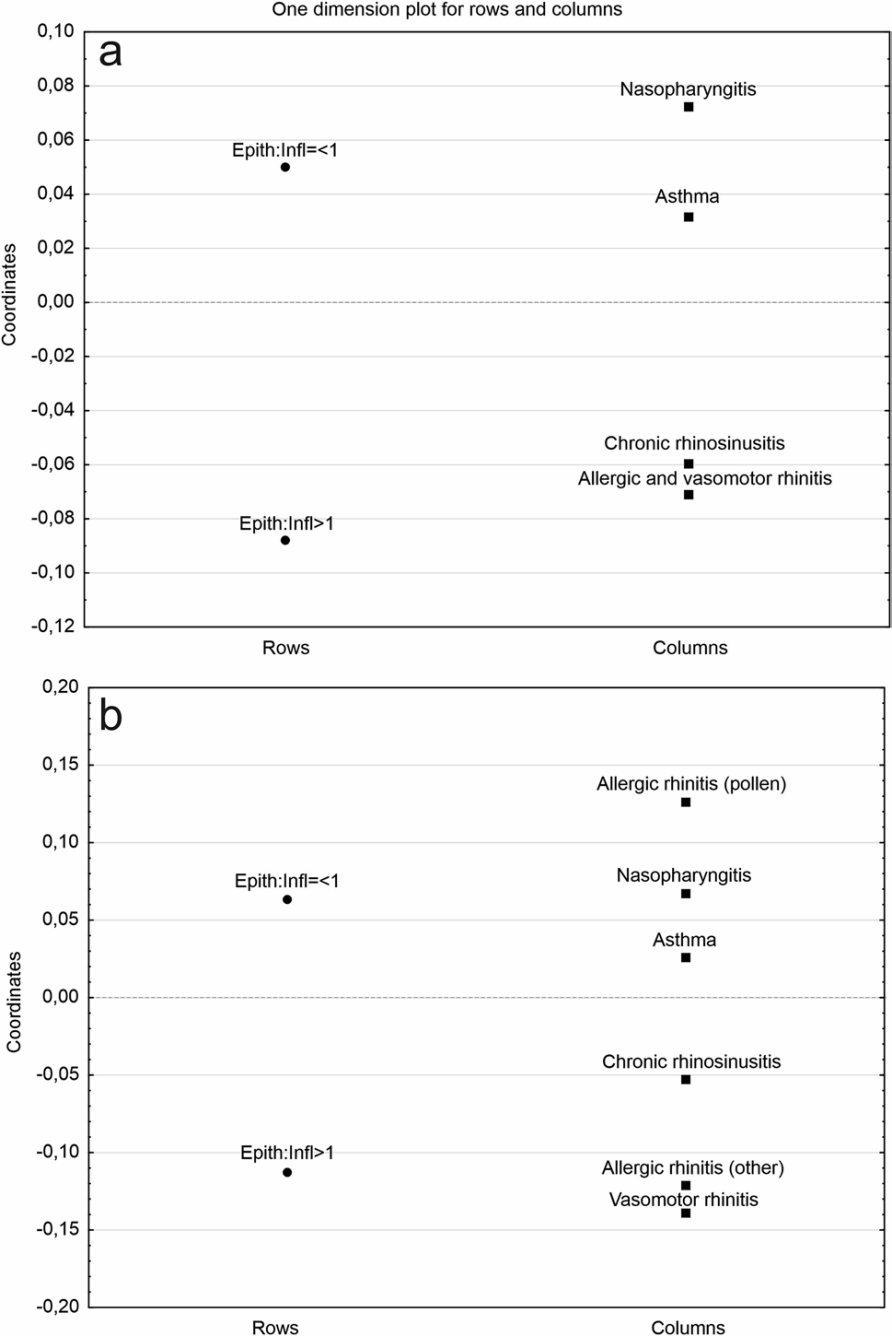
The intensity of nasal inflammation in relation to the primary (**a**) and the more detailed disease entities diagnosis (**b**).

Chi2 test has confirmed the statistically significant relationship between the Epith:Infl ratio and the main, four distinguished groups of diseases (Chi^2^ = 9.6488; p = 0.014). The correspondence analysis (CA) suggests that in patients with chronic nasopharyngitis and asthma, in cytological material the increased inflow of inflammatory cells is more frequently observed (Fig. 1a). When epithelial cells predominate in cytological smears, the allergic and vasomotor rhinitis or chronic rhinosinusitis are diagnosed more frequently, not being closely related to the increased inflammation.

In the next step of the analysis, the more detailed diagnoses were considered. From the group of upper respiratory tract diseases named as allergic and vasomotor rhinitis, three diseases were distinguished (acc. to ICD-10 classification as reported in Material and Methods): allergic rhinitis provoked by pollen, allergic rhinitis provoked by other allergens and vasomotor rhinitis. The statistically significant relationship between the specified diagnoses and the cytological picture was also confirmed (p = Chi^2^ = 22.7708; p = 0.005). In the case of increased inflammation (Epith:Infl ≤ 1), the different chronic problems of nasopharyngitis, including the non-allergic, chronic rhinitis were more often diagnosed, but also allergic rhinitis provoked by pollen allergens was assigned to this group. It clearly indicates that in patients sensitive to pollen allergens, more frequently the increased inflammation is observed, in comparison with the other allergic rhinitis (provoked e.g. by house dust mites, animal epithelia or spores), or vasomotor rhinitis (Fig. 1b).

### Diagnosis versus inflammatory cells

**Figure 2:**
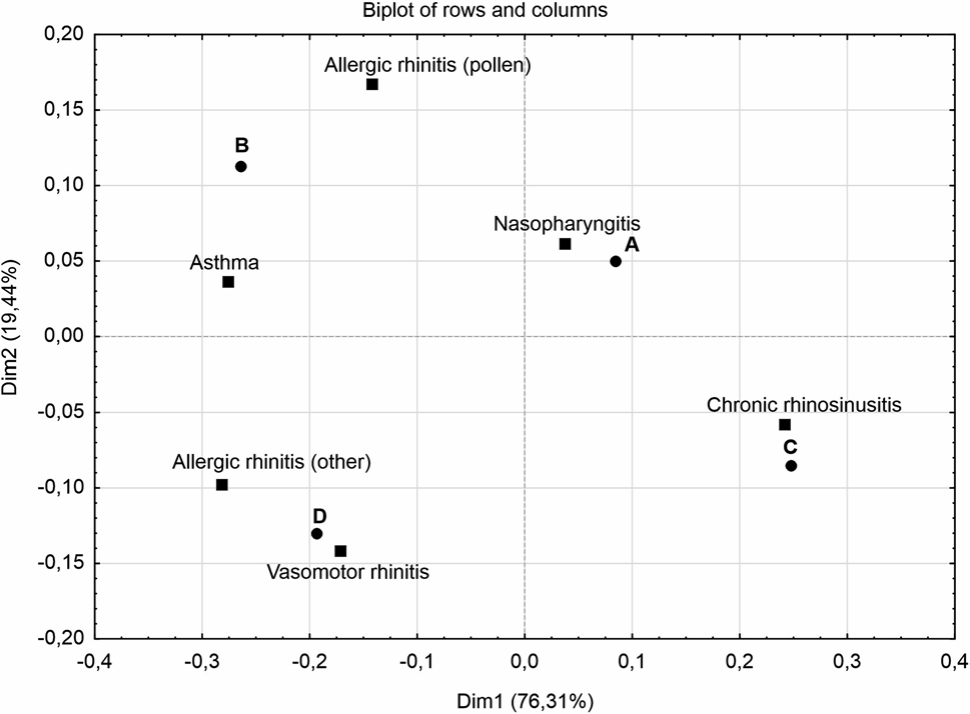
Percentage of patients with an increased level of eosinophils in relation to the nasal inflammation and the diagnosis. The following situations were considered: (A) Epith:Infl ≤ 1 and eosinophils ≤ 1%; (B) Epith:Infl ≤ 1 and eosinophils > 1%; (C) Epith:Infl > 1 and eosinophils ≤ 1%; (D) Epith:Infl > 1 and eosinophils > 1%.

When the percentage of patients with an increased eosinophilic reaction was considered, the statistically significant relationship between diagnosis and the ratio of epithelial and inflammatory cells was found (Chi^2^ = 61.58617; p = 0.000). The cytograms with the elevated content of eosinophils (> 1%) were detected more frequently in patients with the diagnosis of allergic and vasomotor rhinitis (Fig. 2B,D), while patients with chronic nasopharyngitis, manifested eosinophilic inflammation less frequently (situation A), despite the evident migration of the inflammatory cells, similarly to the patients with chronic rhinosinusitis, who, however presented a lower migration cells inflow (situation C) (Fig. 2). These results are compatible with those presented on the Fig. 1b, however contribute much more to explaining the nature of the cellular inflammation state.

**Figure 3:**
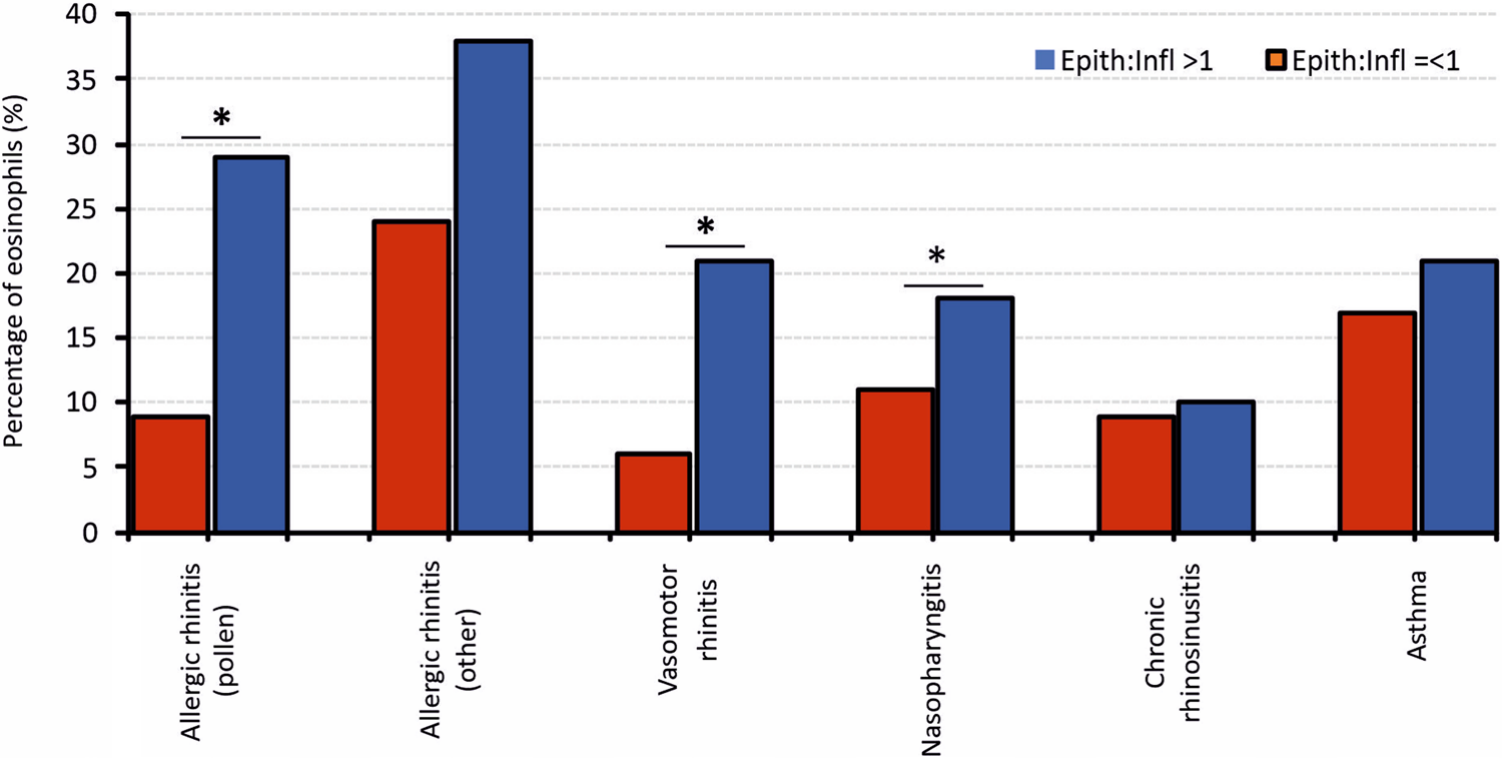
The percentage of eosinophils in the group of inflammatory cells, in patients with/without the signs of inflammation, taking into account the diagnosis. Statistically significant differences were marked (*p < 0.05).

The amount of eosinophils in the group of higher inflammation was significantly lower than in the group of less frequent signs of inflammation (p = 0.000). In relation to the diagnosed diseases, the highest content of eosinophils was found in the case of the other allergic rhinitis, in both studied groups, while the significant differences between the eosinophils percentage were found in case of allergic and vasomotor rhinitis (p = 0.005 and p = 0.000, respectively) and nasopharyngitis (p = 0.02) (Fig. 3). It is clearly seen, that in the case of evident inflammation on the nasal mucosa, the eosinophilic reaction was less intensive. On the other hand, in each of the studied diseases the eosinophilic response is possible, but its intensity is related to the general inflow of the migrating cells provoking the inflammation state in the respiratory tract.

**Figure 4:**
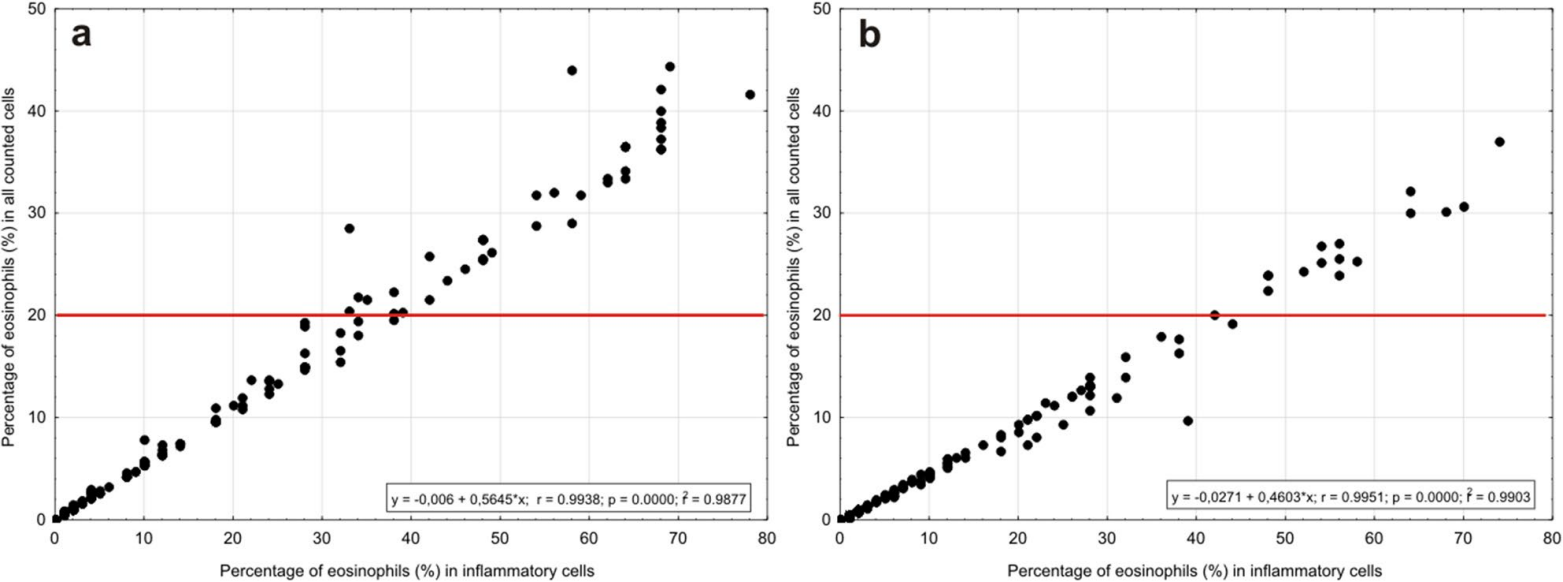
Relationship between percentage of eosinophils counted in all and in inflammatory cells, in (**a**) patients with a normal cytogram (Epith:Infl > 1) and (**b**) with inflammation picture (Epith:Infl ≤ 1). The red horizontal line corresponds with the level of eosinophils in all counted cells, recommended for the diagnosis of non-allergic rhinitis acc. to Heffler et al. (2018)^1^.

The percentage of eosinophils calculated within the group of inflammatory cells is closely related to the percentage of eosinophils among all counted cells (Fig. 4). The value of > 20% of eosinophils in all cells occurring in the nasal mucosa in non-allergic patients is characteristic for non-allergic rhinitis with eosinophilia (NARES), acc. to^1^. This value corresponds in our study to 30–40% of eosinophils in the group of inflammatory cells in patients with a normal cytogram (with no signs of inflammation) and around 45% of eosinophils in patients with the evident inflammatory picture. Considering the fact, that the lower concentration of eosinophils was generally found in the group of the patients with more intense inflammation in cytological smear (Fig. 3), the higher increase in eosinophils appearance within the migrating cells must be achieved in this group to diagnose NARES.

## Discussion

Nasal cytology is a useful diagnostic tool presenting pictorially the current condition of the nasal mucosa in terms of epithelium exfoliation and the inflow of inflammatory cells from the tissue pool^1,20,22^. The information on the local, mucosal inflammation facilitates making a decision on diagnosis and the proposed treatment in patients with allergological, laryngological or other problems of the respiratory tract. It is expected that the cytogram indicates, whether the cytological picture supports the existing inflammation and what kind of inflammatory cells dominate^20^.

Referring to the experience of the Centre of Allergology in Kraków in cytological analyzes we have proposed a new coefficient, which may be an initial criterion for the assessment of cytological material, indicating the intensity of inflammation. As it was shown, the ratio between epithelia and inflammatory cells correlates well with the diagnosis of the selected diseases of upper and lower respiratory tract. Considering two major clinical phenotypes of non-infectious rhinitis: allergic rhinitis (AR) and nonallergic rhinitis (NAR)^23^, NAR, in our study determined as chronic rhinitis, belonging to the nasopharyngitis group, is thought to be closely related to the local, intensive inflammatory process, while AR does not have to be associated with an evident inflow of inflammatory cells, apart from AR provoked by pollen allergens. Discussing the problem of NAR, the authors emphasize, that there is a difference between ICD-10 and ICD-11 classifications, so currently the non-allergic rhinitis is pointed out not in the group of chronic rhinitis, as previously, but in the group of vasomotor and allergic rhinitis and described as an inflammation of nasal mucosa in which allergic mechanisms are not involved and covers many different phenotypes.

We have found, that in about half of the patients with chronic nasopharyngitis, the activity of inflammatory cells is enhanced, while in the rest of patients the persistent inflammation is expected. NAR involves a heterogeneous group of patients suffering from rhinitis without clinical signs of infection and without systemic signs of allergic inflammation, being divided into several sub-groups as follows: drug-induced rhinitis, rhinitis of the elderly, hormonal rhinitis including pregnancy-induced rhinitis, nonallergic occupational rhinitis, gustatory rhinitis, and idiopathic rhinitis^15,24^.

Some authors stated that the higher inflammatory cells migration could be related to more active exposition to the increasing seasonal allergen concentration in intermittent AR, especially plant pollen, e. g. grasses^10^ and the decreasing persistent allergen count in persistent AR^25,26^, provoked by a year-round allergens, e.g. originated from house dust mites^16,26–28^. This observation was confirmed in our study, because when the whole group of patients diagnosed into allergic or vasomotor rhinitis was considered, the less frequent the inflammatory cytograms were obtained. When the diagnoses of allergic and vasomotor rhinitis were analyzed separately, it was clearly stated that among this group of rhinitis, only patients sensitive to pollen allergens manifest the increased influx of migrating cells. Taking into account not only the inflammatory pool cells, but also the relationship between both inflammatory and epithelial cells makes the diagnosis more objective and makes the doctors aware of the depth of the ongoing inflammation. Minimal persistent inflammation, characterized, at nasal cytology, by a presence of neutrophils and a very low ciliated cell expression, was observed in bakers exposed for a long time to flour dust and allowed to diagnose neutrophil, occupational rhinitis^29^.

An in-depth analysis of the cellular composition of cytograms allows for the assessment of inflammation in the context of specific diseases, considering first of all two types of cellular inflammation, provoked by eosinophils and neutrophils. Eosinophils and mast cells are considered to be the central effector cells in the allergic reaction^30^, in the case of NAR, and their occurrence is essential for NAR phenotypes differentiation, especially NARES and NARESMA^1,31^. We have found, that in patients with chronic, non-allergic rhinitis and severe inflammation, the eosinophil-induced response was less pronounced than in patients without intensive inflammatory process, when the other cells contribute to the pathomechanism of reaction.

When AR is assessed by nasal cytology, eosinophils are expected as a predominant cell type, followed by mast cells and basophils^32,33^. In our previous study, we have documented that the number of patients with eosinophils > 20% in the SPT(+) group was higher in patients with persistent symptoms, while in the SPT(-) group, the number of patients with intermittent symptoms in the subgroup > 20% of eosinophils statistically prevailed (p < 0.001)^10^. Tissue infiltration by eosinophils occurs mainly during the late phase of IgE-mediated allergic response and results in the release of eosinophil mediators, which in turn injure the nasal epithelial cells and induce nasal hyperresponsiveness to several irritant stimuli, causing delayed allergic symptoms and perpetuating allergic inflammation^24^.

The increased inflow of eosinophils should be estimated in relation to the intensity of the inflammation process (in our study 11.54% versus 4.89% on average, respectively in Epith:Infl > 1 versus Epith:Infl ≤ 1), even though the other authors reported that the percentage of eosinophils ranges from 20 to 90%^1,3^. We recommend also taking into account that the value of 20% of eosinophils in all counted cells corresponds to 30–40% of these cells in the group of inflammatory cells in patients with normal cytogram and to around 45% of eosinophils in patients with the evident inflammatory picture, when the current disease advancement is estimated.

It should be expected that in patients with less evident eosinophilic reaction, the neutrophils are responsible for the development of inflammation, in a non-specific reaction. This practical information is useful to interpret cellular profiles, while allergic rhinitis and non-allergic rhinitis are suspected. It must be singled out that it is not possible to clearly classify AR as an eosinophilic response and NAR as taking place only on the basis of the nonspecific reaction with the participation of neutrophils (as suggested by Tulic and Hamid^34^). Moreover, chronic and/or untreated AR may result in complications, which include recurrent chronic rhinosinusitis and the formation of nasal polyps, although our results do not indicate the evident inflammation picture in the case of rhinosinusitis. There is a large accumulation of eosinophils and their cytokines in both of these cases, attributed to IL-5 and eotaxin, an eosinophil chemo-attractant, the production of which is significantly increased in the nasal mucosa^34^.

It is worth underlining that patients diagnosed with vasomotor rhinitis as a form of non-allergic inflammation of the nasal mucosa that is characterized by nasal congestion and posterior pharyngeal drainage, present with the cytograms of a less inflammatory design, but the increased eosinophilic reaction (https://icd.who.int/browse11/l-m/en). The pathophysiology of this phenotype is still not fully discovered. Because vasomotor rhinitis, characterized by nasal hyperreactivity, can refer acc. to Gelardi et al. 2022^35^ to both AR and NAR, the occurrence of eosinophils seems to be diagnostically important in patients with excessive reactivity of the nasal mucosa.

The cellular profile of the mucosal inflammation may affect the efficacy of the individualizing of a given patient treatment^31,36^, which is in line with the precision medicine approach. Meltzer et al.^37^ assessed the effect of intranasal fluticasone propionate and beclomethasone dipropionate in seasonal and perennial AR, which showed a favorable therapeutic effect—a decrease in the number of eosinophils and basophils, but a relative lack of change in the percentage of neutrophils. Özgür et al.^38^ used cytology as an objective test of the efficacy of periodic AR treatment with nasal corticosteroid in combination with oral cetirizine, in addition to the subjective symptom scale, reducing the percentage of eosinophils in the mucosa, the number of neutrophils and goblet cells also decreased.

Breaching and penetration of the mucosal barrier is considered crucial for the pathogenesis of chronic rhinosinusitis (CRS), so cytology can help in preliminary differentiation between the types of inflammation. The cells recruited in type 2 (specific for parasites) are mostly eosinophils and mast cells, while neutrophils are more characteristic for types 1 and 3 (specific for viruses and bacteria/fungi, respectively). Patients with type 2 inflammation usually present with a more recalcitrant disease but they may benefit from new therapeutic options including biological treatment (monoclonal antibodies against cytokines)^39^. However, the presented results do not indicate clearly that when rhinosinusitis is diagnosed, the severe inflammatory picture in the nasal smears is observed. In our study asthma was classified as a disease related to more intense inflammation of the nasal mucosa, which probably resulted from the close connection between AR and atopic asthma, and even LAR seems to have a progressive course and constitute a potential risk factor for asthma^15,17,40^.

Gelardi et al. (2009)^4^ proposed to perform nasal cytology as an easy to use diagnostic procedure even for primary care physicians, to indicate, when anti-inflammatory treatments, such as intranasal corticosteroids and subcutaneous or sublingual allergen immunotherapy, are needed to be ordered. The ratio proposed by us can be helpful in the estimation of inflammation in poly- and mono-sensitized patients, defining a distinct AR phenotype as suggested by Gelardi et al.^41^, who showed a more intense inflammatory infiltrate in poly- than in mono-allergic patients. In some doubtful situations, nasal cytology can be used to assess local response in a challenge test, e. g. indicating patients with LAR formerly defined as nonallergic rhinitis with eosinophilia syndrome (NARES), characterized by large numbers (inconsistently defined as > 5% to > 20%) of eosinophils on nasal smear^18^.

Nasal cytology may be useful not only in differentiating infectious from non-infectious nasal and/or sinus disease, but also in overlapping (mixed) rhinitis^42^. For example, neutrophils may be present not only in acute and chronic rhinosinusitis, but also in conjunction with eosinophils in patients with allergic rhinitis who also have an acute infection process. As Gelardi et al.^4^ suggested, in some patients, clinical symptoms may be result from a different pathomechanism of inflammation, including nonallergic non-infectious rhinitis overlapping AR. It should be stressed, that in patients with less evident eosinophilic reaction, the neutrophils are responsible for the development of inflammation, in a non-specific reaction. When this response is expected, it is useful to interpret cellular profiles, while the allergic rhinitis and non-allergic rhinitis are suspected.

## Conclusions

1. While the nasal cytograms are assessed, the relation between inflammatory and epithelial cells should be considered, as closely related to the disease entity. Particularly, patients with chronic nasopharyngitis, asthma and allergic rhinitis with pollen sensitivity frequently present in cytological material the increased inflow of inflammatory cells. The information about the ratio between epithelial and inflammatory cells makes the diagnosis more comprehensive.
2. The increase of the percentage of eosinophils in the patients with an evident inflammation on the nasal mucosa is significantly lower in comparison to those who presented less evident inflammation in the cytological pictures.
3. In the case of evident inflammation on the nasal mucosa, the eosinophilic reaction was less intensive, however considering each of the studied disease the eosinophilic response is possible, but its intensity is related to the general inflow of the migrating cells provoking the inflammation state in the respiratory tract. The higher amount of eosinophils was found in case of allergic rhinitis.
4. The share of eosinophils in the entire pool of cells corresponds in direct proportion to their share in the pool of inflammatory cells. When the percentage of eosinophils in the group of migrating cells oscillates around 45%, the diagnosis of non-allergic rhinitis with eosinophilia syndrome is highly probable in patients with more intense inflammation, while in the patients with the weaker signs of inflammation, the lower value of the eosinophil concentration is expected, if NARES is diagnosed.

## Material and methods

Retrospective analysis of the results of exfoliative cytology was carried out in a group of 842 patients diagnosed with different diseases of the respiratory tract (URT) at the University Hospital in Kraków (533 female, 309 male; aged 40.17 ± 17.17 years), in 2017–2019. Referring to the study project, accepted by the Ethical Commission of the Jagiellonian University, the criteria of patients inclusion were as follows: (1) nasal exfoliative cytology test performed as part of standard allergological or laryngological diagnostics; (2) age ≥ 18 years. For the study purposes, the following groups of respiratory tract diseases were distinguished in accordance with the International Statistical Classification of Diseases and Related Health Problems (ICD) (https://icd.who.int/browse10/2019/en#/J30-J39) version-10: (1) diseases of upper respiratory tract (URT), including (1) vasomotor and allergic rhinitis with an indication on: allergic rhinitis provoked by pollen allergens, other allergic rhinitis and vasomotor rhinitis (named: allergic and vasomotor rhinitis); (2) chronic rhinitis and chronic nasopharyngitis (named: nasopharyngitis); (3) chronic rhinosinusitis and bronchial asthma (named: asthma) as a lower respiratory tract disease.

Cytology was performed in accordance with the order for allergological and laryngological indications in patients diagnosed most of all into different diseases of the upper respiratory tract (URT). Nasal smears were obtained using the scraping cytology^1,3,6^, after prior discontinuation of appropriate drugs (antihistamines for 1 week, local steroids for 2 weeks and systemic steroids up to 4 weeks before the examination). Patients cleaned the nostrils in order to remove excess secretions. Immediately before taking the smear, the appearance of the nasal mucosa, its color, presence of edema, type and amount of discharge were assessed.

Cytological material was collected with a sterile, plastic bacteriological loop from the nasal mucosal surface of the inferior turbinate from its middle part, from both nostrils and placed on a basic, microscope slide. Slides were stained with the standard May-Grünwald-Giemsa (MGG) method and recognized and counted under optical microscopy at 1000× magnification with oil immersion (Axioskop-2, Carl Zeiss Jena, Germany).

The quantitative analysis of the cellular material was performed based on the own procedure of the University Hospital described by Myszkowska^3^ following the Polish indications^43^ and the recommendations published by Gelardi et al.^20^ and Heffler et al.^1^. Both inflammatory and epithelial cells were counted in the recommended 50 fields of view (up to 500 cells, minimum 200 to consider the sample as adequate). The count of each cell type was expressed in the cytograms as a percentage of the total cells, and as a percentage of cell type within a group of inflammatory and epithelial cells, separately. The ratio of the amount of epithelial cells to inflammatory cells (Epith:Infl) was calculated and shown as two ranges, ≤ 1 and > 1. The values below or equal 1 refer to the increased infiltration of inflammatory cells causing the inflammation, while the dominance of epithelial cells is related to the normal physiological state^44^. The proposed ratio was used to analyze the relation between the obtained cytograms and the diagnosis in accordance with the International Statistical Classification of Diseases and Related Health Problems (ICD) (https://icd.who.int/browse10/2019/en#/J30-J39), specifically in the case of the increased percentage of eosinophils in the cytological material.

We confirm that all methods were carried out in accordance with relevant guidelines and regulations. The study was approved by the Jagiellonian University Bioethics Commission (No 1072.6120.88.2022, dated 21 Apr 2022), which waived the need for informed consent due to study character, qualified as not being the medical experiment. In accordance with the Jagiellonian University Bioethics Commission guidelines the retrospective study has to be reviewed by the Commission, but does not require the attachment of information for the study participant, the participant's consent form and the consent form for the processing of personal data (https://kbet.cm.uj.edu.pl/przygotowanie-wniosku-do-komisji/jakie-badania-nie-maja-charakteru-eksperymentu-medycznego/).

The results were analyzed according to the standard statistical methods. In the first step the normality of the distribution was tested using the Shapiro–Wilk test. The Mann–Whitney U test was applied to compare the percentage of eosinophils in two independent groups of samples (Epith:Infl > 1 vs. Epith:Infl ≤ 1, while the Kruskal–Wallis ANOVA was used in the case of more than two independent samples (eosinophilic reaction in relation to inflammatory response in 4 distinguished subgroups: (A) Epith:Infl ≤ 1and eosinophils ≤ 1%; (B) Epith:Infl ≤ 1and eosinophils > 1%; (C) Epith:Infl > 1and eosinophils ≤ 1% and (D) Epith:Infl > 1and eosinophils > 1%. The value of 1% of the eosinophils in the inflammatory cells pool has been chosen, as recommended value observed the physiological condition (in healthy individuals)^45^. Chi^2^ test and the correspondence analysis (CA) were applied to check the relations between intensity of inflammation, the eosinophilic reaction and the disease entities. For all these statistical tests the statistical significance was accepted at the level of α ≤ 0.05. Statistical analysis was performed using the Statistica program version 13.0 (StatSoft, Inc. 1984–2013).

### Ethics approval

The study was approved by the Jagiellonian University Bioethics Commission (No 1072.6120.88.2022, dated 21 Apr 2022), which waived the need for informed consent due to study character, qualified as not being a medical experiment. In accordance with the Jagiellonian University Bioethics Commission guidelines the retrospective study has to be reviewed by the Commission, but does not require the attachment of information for the study participant, the participant's consent form and the consent form for the processing of personal data (https://kbet.cm.uj.edu.pl/przygotowanie-wniosku-do-komisji/jakie-badania-nie-maja-charakteru-eksperymentu-medycznego/). Neither during the analyzes, nor during the preparation of the manuscript any patients personal data were presented.

## Data Availability

All patients data and the results of the cytology have been taken from the medical data base of the University Hospital in Kraków, available for the only hospital workers according to the special permission. As the patients data are saved in the data base, their further dissemination is prohibited by the hospital management. The datasets generated and/or analysed during the current study are not publicly available due to the patient data protection, but are available from the corresponding author on reasonable request.
